# Practical Issues in Imputation-Based Association Mapping

**DOI:** 10.1371/journal.pgen.1000279

**Published:** 2008-12-05

**Authors:** Yongtao Guan, Matthew Stephens

**Affiliations:** 1 Department of Human Genetics, University of Chicago, Chicago, Illinois, United States of America; 2 Department of Statistics, University of Chicago, Chicago, Illinois, United States of America; University of California San Diego and The Scripps Research Institute, United States of America

## Abstract

Imputation-based association methods provide a powerful framework for testing untyped variants for association with phenotypes and for combining results from multiple studies that use different genotyping platforms. Here, we consider several issues that arise when applying these methods in practice, including: (i) factors affecting imputation accuracy, including choice of reference panel; (ii) the effects of imputation accuracy on power to detect associations; (iii) the relative merits of Bayesian and frequentist approaches to testing imputed genotypes for association with phenotype; and (iv) how to quickly and accurately compute Bayes factors for testing imputed SNPs. We find that imputation-based methods can be robust to imputation accuracy and can improve power to detect associations, even when average imputation accuracy is poor. We explain how ranking SNPs for association by a standard likelihood ratio test gives the same results as a Bayesian procedure that uses an unnatural prior assumption—specifically, that difficult-to-impute SNPs tend to have larger effects—and assess the power gained from using a Bayesian approach that does not make this assumption. Within the Bayesian framework, we find that good approximations to a full analysis can be achieved by simply replacing unknown genotypes with a point estimate—their posterior mean. This approximation considerably reduces computational expense compared with published sampling-based approaches, and the methods we present are practical on a genome-wide scale with very modest computational resources (e.g., a single desktop computer). The approximation also facilitates combining information across studies, using only summary data for each SNP. Methods discussed here are implemented in the software package BIMBAM, which is available from http://stephenslab.uchicago.edu/software.html.

## Author Summary

Genotype imputation is becoming a popular approach to comparing and combining results of multiple association studies that used different SNP genotyping platforms. The basic idea is to exploit the fact that, due to correlation among untyped and typed SNPs, genotypes of untyped SNPs in each study can be inferred (“imputed”) from the genotypes at typed SNPs, often with high accuracy. In this paper, we consider several issues that arise when applying these methods in practice, including factors affecting imputation accuracy, the importance of taking account of imputation uncertainty when testing for association between imputed SNPs and phenotype, how imputation accuracy affects power, and how to combine results across studies when only single-SNP summary data can be shared among research groups.

## Introduction

Ongoing large-scale genetic association studies, in an attempt to identify variants and genes affecting susceptibility to common diseases, are typing hundreds of thousands of SNPs in thousands of individuals, and testing these SNPs for association with phenotypes. Although this is a large number of SNPs, an even larger number of SNPs remain untyped. For example, the International HapMap Project contains genotype data on more than 3 million SNPs [1], many of which will not be typed in current studies. Imputation-based association analysis [2],[3] provides a powerful framework for testing these untyped variants for association with a phenotype.

The idea behind these imputation-based approaches is to exploit the fact that untyped SNPs are often correlated with typed SNPs, so genotype data on typed SNPs can be used to indirectly test untyped SNPs for association with phenotypes. Specifically, the approaches in [2],[3] exploit available information about patterns of correlation among typed and untyped SNPs in a reference *panel* of densely-genotyped individals (e.g. the HapMap samples) to explicitly predict, or “impute”, the genotypes at untyped SNPs in a study sample, and then test these imputed genotypes for association with a phenotype, taking account of uncertainty in the imputed genotypes. We emphasise this last point, because imputation could be interpreted as testing a single estimate of the genotypes (as in [4], for example), ignoring uncertainty, whereas we take a broader interpretation, to include methods that incorporate uncertainty in imputed genotypes.

Testing untyped variants via imputation both increases power to detect associations [2],[3] and provides improved explanations for detected associations, for example by helping to identify the most plausible causal variant or variants [2]. Imputation also provides a powerful framework for combining information across multiple association studies performed on different genotyping platforms, since it allows each study to test the same set of SNPs regardless of the genotyping platform used (e.g. [5],[6]).

Here we focus on several important issues that arise when applying imputation-based methods in practice. These include i) factors affecting imputation accuracy, including choice of reference panel; ii) the effects of imputation accuracy on power to detect associations; iii) the relative merits of Bayesian and frequentist approaches to testing imputed genotypes for association with phenotype; and iv) how to quickly and accurately compute Bayes factors for testing imputed SNPs.

We find that imputation-based methods can be relatively robust to imputation accuracy: small changes in imputation accuracy produce small changes in power to detect associations, and even when average imputation accuracy is poor (as could occur if the panel is not well-matched to the study sample), imputation-based approaches can still improve power compared with no imputation. Comparing frequentist and Bayesian methods for testing imputed SNPs, we note that ranking SNPs by their *p* value from a likelihoood ratio test gives the same results as a Bayesian procedure based on a particular prior assumption–specifically that difficult-to-impute SNPs tend to have a larger effect on phenotype. We argue that since this assumption is unnatural, alternative Bayesian approaches that do not make this assumption should have an advantage, in principle, for testing untyped SNPs. We illustrate the practical effect of this advantage via simulation experiments. Within the Bayesian framework we find that good approximations to a full analysis can be achieved by simply replacing unknown genotypes with a point estimate, their posterior mean. This approximation produces results that are more accurate, and orders of magnitude faster to compute, than sampling-based estimates used by [2],[3]. Indeed, the resulting approximation is faster, even, than application of standard frequentist methods in this context, and the methods we discuss are fast enough to be practical on a genome-wide scale with very modest computational resources (e.g. a single desktop computer). The accuracy of this approximation also has helpful implications for combining information across studies in situations where only single SNP summary data can be easily shared.

### Imputation-Based Association Mapping

We now describe more formally the imputation-based approaches to association mapping that we take here, and introduce notation for use in later sections. Readers whose primary interest is in our practical findings may wish to skip the Results section, and refer back to this section for reference.

The imputation-based approach we take here is based on using a “prospective” model relating phenotypes to genotypes, and so is appropriate for analyzing phenotypes and genotypes on individuals sampled randomly from a population. We consider its applicability to other designs, such as case-control studies, in the Discussion. (See [4] for related work based on a retrospective likelihood.)

Let *Y* denote measured phenotype values for *n* randomly-sampled individuals on whom genome-scan genotype data *G* have been collected. We use *y_i_* to denote the phenotype of individual *i*, and g*_ij_* for the genotype of individual *i* at SNP *j* (coded as 0, 1 or 2 copies of the minor allele), with g_·*j*_ denoting the vector of genotypes at SNP *j*. We also assume the availability of denser genotype (or haplotype) data, *H*, on a “panel” of unrelated, unphenotyped, individuals. In our examples this panel will consist of subsets of individuals from the International HapMap Project. For notational simplicity we assume that the columns of *H* and *G* are augmented, as necessary, so that they refer to the same set of SNPs (i.e. so they have the same number of columns, with the *j*th column of each referring to the same SNP *j*). Thus, if a SNP *j* is typed in *H* but not in *G* then g_·*j*_ will consist of all “missing” observations.

The aim of our imputation-based mapping approach is to assess whether a SNP, *j*, that is genotyped in *H* but not in *G*, is associated with the phenotype *Y*. Let *β* denote the parameters in a model that relates phenotypes *Y* with genotypes g_·*j*_ at SNP *j*. To obtain a likelihood for *β*, given the observed data *Y*, *G*, *H*, we take the standard regression-based approach of conditioning on the genotypes *G*, *H*, and using the conditional distribution *p*(*Y*|*G*, *H*, *β*) as the likelihood. This approach implicitly assumes that the genotypes alone contain no information about *β* (or, at least, it ignores any such information). [Although in principal the genotypes alone *could* provide information about whether a SNP has an effect on phenotype (e.g. through signatures of selection), such information seems likely to be both limited, and difficult to extract.] Under this assumption the likelihood for *β* can be written as
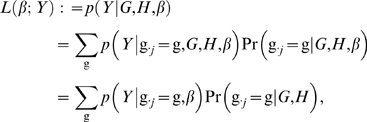
(1)where the last line invokes the assumption that the genotype distribution does not depend on *β*.

From Expression (1) we see that, for an untyped SNP, the likelihood is a weighted average of the complete data likelihood (i.e. the likelihood if the genotypes at SNP *j* were observed to be g_·*j*_) over all possible values of g_·*j*_. The weights, Pr(g_·*j*_ = g|*G*, *H*), are determined by the distribution of the unobserved genotypes given the observed genotypes (which can be obtained in several ways; here we use methods from [7]). This sum has too many terms to be computed directly. However, if the two distributions that occur in each term of this sum are independent across individuals, as they will be in settings considered here, then the likelihood simplifies to a computationally-tractable form:
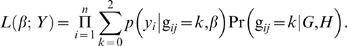
(2)


We will compare two different approaches to using this likelihood to test the null hypothesis *H*_0_ that SNP *j* is unassociated with phenotype, versus the alternative hypothesis *H*_1_ that SNP *j* is associated with phenotype. The first approach is to use the generalized likelihood ratio test statistic,
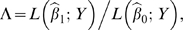
(3)where *βˆ*_0_ and *βˆ*_1_ denote the maximum likelihood estimates of *β* under *H*_0_ and *H*_1_ respectively. Under standard theory, 2log(Λ) has an asymptotic *χ* ^2^ distribution under *H*_0_.

The second approach we consider is the Bayesian approach from [2]. In Bayesian statistics, the strength of the evidence for *H*_1_ vs *H*_0_ is given by the Bayes factor for *H*_1_ vs *H*_0_, defined as
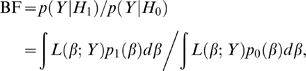
(4)where *p*_0_ (·) and *p*_1_ (·) denote prior distributions on *β* under *H*_0_ and *H*_1_. Note that this expression for the Bayes factor bears some resemblance to the likelihood ratio Λ, but whereas in Λ the numerator and denominator are maximised with respect to unknown parameters *β*, in the Bayes factor they are *integrated* with respect to these parameters, weighted by the prior (which has the effect of averaging over plausible values of the parameters). Large values of the Bayes factor indicate evidence for *H*_1_ over *H*_0_, whereas small values indicate evidence for *H*_0_ over *H*_1_. Bayes factors have a number of general advantages over *p* values as measures of evidence [2],[8],[9], and, as we show below, a particular advantage, at least in principle, when testing untyped SNPs.

For a typed SNP at which genotypes are observed to be g, the Bayes factor, BF(g), can sometimes be computed analytically (see below). For an untyped SNP the Bayes factor is the weighted average of BF(g) over all possible values for g. Indeed, substituting the likelihood (1) into the numerator of (4) gives
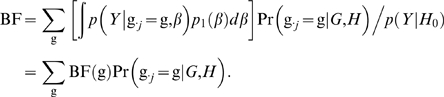
(5)Since this sum typically has too many terms to be computed directly, it must be approximated by computational methods; we compare three different methods later in this paper.

### Example: Normal Phenotype

We now give explicit expressions for the likelihood and Bayes factor in the case of a continuous (quantitative) phenotype *Y*, which will be our primary focus for the rest of this paper. We assume a normal linear model relating *Y* to the genotypes g_·*j*_ at a SNP *j* of interest:
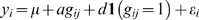
(6)where *μ* is the phenotype mean for individuals with g*_ij_* = 0; *a* and *d* represent, respectively, an additive and dominance effect for SNP *j*; **1**(*A*) is the indicator function, taking value 1 if *A* is true, and 0 otherwise; and *ε_i_* are independent and identically distributed *N*(0,1/*τ*) error terms, where 1/*τ* represents the variance of *Y* within each genotype class. Thus the parameters in this model are *β* = (*μ*, *a*, *d*, *τ*), and the null hypothesis *H*_0_ is *a* = *d* = 0.

Under this model, the likelihood (2) becomes, for each *y_i_*, a mixture of three normal distributions:
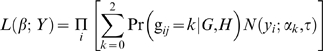
(7)where *N*(·;*α*, *τ*) denotes the normal density with mean *α* and variance 1/*τ*, and *α*_0_ = *μ*, *α*_1_ = *μ*+*a*+*d*, *α*_2_ = *μ*+2*a*.

To compute Bayes factors, we use a prior based on prior D2 from [2]. Among other assumptions, this prior assumes that *a* and *d* are independent and normally distributed, with mean 0, and respective variances 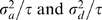. As noted by [2], the assumption that *a* and *d* are a priori independent is not ideal, since one might expect *a* and *d* to be dependent. However, dependence can easily be introduced by averaging results from prior D2 over several values of *σ_a_* and *σ_d_*, as we do here. Furthermore, in simulations, [2] found that the results from this prior generally agreed well with results from another prior, D1, based on more realistic assumptions.

Under this prior, the Bayes factor for a SNP with genotypes g, BF(g), can be computed analytically ([2], Protocol S1):
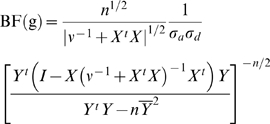
(8)where *Y̅* is the mean of the phenotypes *Y*; *ν* ^−1^ is the 3×3 diagonal matrix with diagonal elements 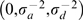; and *X* is an n×3 design matrix, which is a function of the genotypes g. Specifically, the first column of *X* is a vector of 1s, the second column is the vector of genotypes g, and the third column is a vector of indicators for whether the genotypes are heterozygotes (*X_i_*_3_ = **1**(*g_i_* = 1)).

To compute the likelihood ratio (3) and Bayes factor (5) we now need two things. First, we need expressions for Pr(g_·*j*_ = g|*G*, *H*); that is, we need to have ways to estimate the (distribution of) genotypes at SNP *j* from the observed genotype data *G*, *H*. Second, because the sum (5) is over all possible genotypes at SNP *j*, and therefore typically contains a very large number of terms, we need efficient computational methods for approximating this sum. (In contrast, Λ can be obtained relatively easily by numerical optimisation of (2) over *β*.) The first two sections of the results, Genotype Imputation, and Bayes Factor Calculation, deal with each of these two issues in turn. Subsequent sections deal with comparisons of the use of the Bayes factor and Λ to detect associations with phenotypes, and the effects of imputation accuracy on power to detect associations.

## Results

### Genotype Imputation

While there are many possible approaches to predicting unknown genotypes from patterns of LD [e.g. 10],[11],[12], both [2],[3] use similar methods based on Hidden Markov models: the PAC model from [13] implemented in software PHASE and IMPUTE, and the cluster-based model from [7] implemented in software fastPHASE and BIMBAM. In comparisons in [7] the two models produced very similar accuracy for imputed genotypes, and were more accurate than other methods considered. Both models also produced approximately calibrated predictions (e.g. genotypes assigned a probability of 90% by these models were correct in approximately 90% of cases). Here we focus on the cluster-based model from [7] because it has certain computational and practical advantages over the PAC model (e.g. it can deal easily with unphased panel data). However, we expect many of our conclusions to apply more generally (e.g. see Discussion).

In brief, the model from [7] assumes that each SNP along each sampled haplotype has an (unknown) cluster membership that changes, in a Markovian way, along the chromosome. Conditional on cluster memberships, alleles are sampled independently from cluster-specific and SNP-specific allele frequencies, *θ*. Other parameters of the model include the jump rates for cluster memberships, *r*; and the probabilities of jumping to each cluster, *α*. In the most general version of the model, which we consider here, both *r* and *α* are allowed to vary along the genome; see [7] for full details. [7] give an EM algorithm for estimating the parameters (*θ*, *α*, *r*) from either phased or unphased genotype data, and describe how the model can be used to impute missing genotypes and estimate haplotypic phase. For example, given parameter estimates (*θ̂*, *α̂*, *r̂*), the distribution of a missing genotype in individual *i* at SNP *j* can be approximated as Pr(g*_ij_* = g|g*_i_*_·_,*θ̂*,*α̂*,*r̂*) where g*_i_*_·_ is the vector of observed genotypes in individual *i*.

[7] compared several approaches to genotype imputation using data with genotypes missing at random. In their comparisons the cluster-based model provided more accurate imputed genotypes than other methods provided that i) the number of clusters was sufficiently large, and ii) predictions of genotype probabilities were averaged across multiple applications of the EM algorithm. Here we examine factors affecting the accuracy of genotypes imputed using this model in the context of imputation-based association mapping, where patterns of missingness are highly structured, not random. We took a genotype data, *G*, on chromosome 22 from 675 Caucasian individuals enrolled in the PRINCE study [14] who have been genotyped on the Illumina 317k chip as part of an ongoing genome-wide association study to study statin response and lipid-related phenotypes. To provide a set of test SNPs on which to assess imputation accuracy we masked all 675 genotypes at every 25th SNP (masking 220 SNPs in each trial, this procedure being repeated 5 times by shifting the starting SNP, so masking 1100 SNPs in total). We then assessed various strategies for using the cluster-based model to impute the masked genotypes, using subsets of the HapMap data as a panel, *H*. Except where noted, results here are based on using the 60 HapMap CEU (European) parents as the panel. We assessed accuracy of imputed genotypes by comparing the true genotypes with the “best guess” imputed genotypes (i.e. the genotype assigned the highest probability) and computing the genotype error rate, being the proportion of best guess genotypes that are incorrect.

#### Methods of Parameter Estimation

Our first, most striking, finding is that imputing genotypes using parameter estimates obtained by maximising the full likelihood *L*(*θ*, *α*, *r*; *G*, *H*) = Pr(*G*, *H*|*θ*, *α*, *r*) performs much less well than imputing genotypes using parameter estimates obtained by maximising the likelihood for *H* only, *L*(*θ*, *α*, *r*; *H*) = Pr(*H*|*θ*, *α*, *r*). For example, when using the CEU HapMap parents (phased data) as a panel, the genotype error rates for these two strategies were, respectively, ∼12% vs ∼6%. The poor performance of the former strategy is not simply due to the EM algorithm getting stuck in poor local modes in the likelihood surface for the larger data set: after first fitting the model to *H* only, applying additional iterations of the EM algorithm with the full data *G*, *H* also worsened imputation accuracy (data not shown).

These results may appear initially counter-intuitive. However, we note that in the available data, only *H* contains information about patterns of LD between typed and untyped SNPs, which is the relevant information when using typed SNPs to impute untyped SNPs. For example, if genotypes at SNPs *A* and *B* are predictive of genotypes at a SNP *C*, and *A* and *B* are typed in the cohort, but *C* is untyped, then all that matters for predicting genotypes at *C* is the conditional distribution Pr(g*_C_*|g*_A_*,g*_B_*) (in an obvious notation); in particular, information on LD between *A* and *B* is irrelevant. The cohort data *G*, which contain data on only the typed SNPs *A* and *B*, do not contain information about this conditional distribution, and so including *G* in the model fit cannot improve imputation accuracy at *C*. This explains why including *G* in the model fit does not improve imputation accuracy, and also supports the strategy used by [3], who perform imputation for each cohort individual independently conditional only on the panel data. It does not however fully explain why including *G* in the model fit actually *worsens* imputation accuracy. A possible explanation for this is that fitting the model to *G* and *H* effectively reduces the number of clusters available to model *H*, since some are “stolen” by the large number of individuals in *G*, and it is this effective reduction in the number of clusters that worsens imputation accuracy. (Note that one might expect including *G* in the model fit to improve imputation accuracy at sporadically missing genotypes at typed SNPs in *G*—as opposed to systematically missing genotypes at untyped SNPs that are the main focus of imputation—since here LD among typed SNPs is what matters, and *G* contains information on this. In separate experiments (results not shown) we did observe that fitting the model using (*G*, *H*) produced a small improvement in this type of imputation accuracy compared with fitting it to *H* alone.)

Besides increasing imputation accuracy at untyped SNPs, fitting the model to *H* alone has the happy consequence of considerably reducing computational expense, particularly in studies with many individuals. This computational saving is due both to the reduced number of individuals in *H* compared with *H*, *G*, and to the fact that *H* can often be assumed phase-known (computational cost for phased data is linear in *K*, whereas for unphased data it is quadratic in *K*).

#### Number of Clusters and EM Iterations

We found that accuracy of imputed genotypes improved with increased number of clusters (*K*), and with the number of EM runs over which results were averaged (*E*); see Table 1. However, differences in genotype accuracy across different values of these parameters were relatively minor (error rates all lay in the range 6.2%–7.3%). Substantially increasing the number of clusters, to 100, produced essentially the same accuracy as using 30 clusters (data not shown). We also experimented with increasing the number of iterations per EM run to improve convergence of parameter estimates during the model fitting procedure, but this did not consistently improve accuracy (error rates typically changed by less than 0.1%, sometimes increasing, sometimes decreasing).

**Table 1:** Error rates (proportion of cases where the most probable genotype does not match the true genotype) for different genotype imputation strategies, using the model of [7] and the (phased) CEU HapMap parents as a panel.

Number of Clusters	Number of EM runs	Error Rate
K = 10	E = 5	0.073
K = 10	E = 10	0.072
K = 10	E = 20	0.069
K = 20	E = 5	0.068
K = 20	E = 10	0.064
K = 20	E = 20	0.064
K = 30	E = 5	0.064
K = 30	E = 10	0.063
K = 30	E = 20	0.062

We use *K* to denote the number of clusters used, and *E* to denote the number of EM runs over which genotype probabilities are averaged. In each case we used 10 iterations of the EM algorithm in each run.

#### Comparison with IMPUTE

We compared imputation accuracy with that of the PAC model, used by [3], implemented in the software IMPUTE. For these data IMPUTE produced an error rate of 6.6% (X. Wen, personal communication), very similar to the results from our approach, confirming the finding from [7] that the two models produce very similar genotype accuracy, at least provided *K* in the cluster-based model is set appropriately, and the cluster-based model is fitted only to *H*. (Note that the data we consider here come from chromosome 22, which has an above-average recombination rate, and hence below-average linkage disequilibrium. This is expected to make imputation harder than in typical genomic regions, and may partly explain why error rates reported here are slightly higher than in [3].)

#### Unphased Panel

One advantage of the cluster-based model over the PAC model is that it can easily deal with the situation where the panel of densely-genotyped individuals is unphased, whereas the PAC model is much harder to adapt to that setting (e.g. IMPUTE assumes that a phased panel is available). Since the HapMap provides many accurately phased individuals, this advantage will not always be important. However, it does make it easier to exploit any denser unphased genotype data that may be available in some cases (e.g. from resequencing of a candidate region). Motivated by this, we examined the effect of having an unphased panel, by performing imputation with the model fit to the *unphased* CEU HapMap parents. Compared with having a phased panel, the error rates for the unphased panel were consistently a few tenths of a percent higher (e.g. 7.6% vs. 7.2% for E = 10, K = 10).

#### Mismatched Panel

We examined the effect of having a panel that is not well matched to the sample, by using the African (YRI) and Asian (CHB+JPT) HapMap samples as a panel to impute genotypes in the European individuals. We found that using either of these panels individually substantially increased error rates (17% for CHB+JPT, 25% for YRI). However, using a combined panel of CEU+YRI+CHB+JPT gave an error rate only slightly higher than using CEU alone (7.8% for combined panel vs. 6.2–7.3% for CEU). This demonstrates that imputation accuracy can be relatively robust to mismatches between the panel and cohort samples, provided that the panel contains at least some individuals with genetic variation representative of the cohort. For intuition into why this happens, note that to impute an individual's genotypes, the cluster-based model (and, indeed, the PAC model) attempts to explain the individual's observed genotypes using a mosaic of panel haplotypes. This can work provided the panel contains suitable haplotypes, even if the panel also contains a large number of unsuitable haplotypes. Thus, although the issue of panel choice merits further study, using the combined panel should be a helpful strategy when imputing genotypes in a cohort that may not be well represented by a single HapMap analysis panel (e.g., admixed individuals).

#### Trade-Off between Accuracy and Call Rate

**Figure 1:**
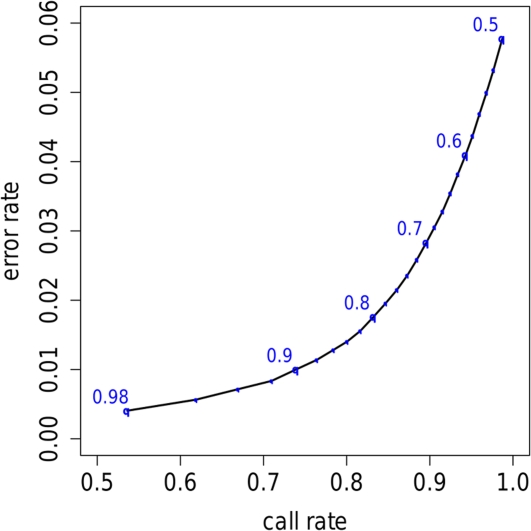
Graph showing the trade-off between call-rate and error rate, as the probability threshold for calling an imputed genotype is varied. Numbers along the line indicate the thresholds that produce the corresponding call rate and error rate; small black points indicate results for intermediate thresholds in increments of 0.02. For example, if we call only those imputed genotypes assigned probability >0.9 of being correct, then approximately 74% of imputed genotypes are called, and of these called genotypes approximately 1% are incorrect.

The error rates above are average error rates across all SNPs, assuming *all* genotypes are called (i.e. 100% call rate). Both the PAC and cluster-based models provide, for each genotype, a probability for each of the three possibilities. If we consider only those genotypes assigned a high probability then error rates can be substantially reduced, at the expense of reduced call rate. For example, if we call only those genotypes assigned a probability of at least 90% then the error rate is reduced to 1%, with a call rate of 74%. Figure 1 shows the correspondance between call rate and accuracy for these data. Although we have used different data, and different approaches to imputation, the results are rather similar to the analogous results in [3] (their Figure 1).

### Bayes Factor Calculations

The Bayes factor for an untyped SNP (Equation (5)) involves a sum over a very large number of terms, and it is computationally impractical to compute this sum directly. In practice then we must use methods to approximate this Bayes factor. Here we compare three different approaches to making this approximation.

The first appproach is the “naive” Monte Carlo estimator used by both [2],[3], given by:
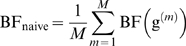
(9)where g^(1)^,…,g^(*M*)^ are independent and identically-distributed samples from *Pr*(g_·*j*_|*G*, *H*). For sufficiently large *M* this estimator will give an accurate approximation to the BF (5). More precisely, it converges to the true value as *M*→∞. However, in practice we have found that for moderate values of *M* ( = 1,000 say) this estimator can have a large standard deviation, producing unreliable estimates for some SNPs (see below).

The second approach is based on an importance sampling estimator [e.g. 15]:
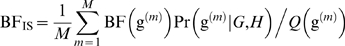
(10)where g^(1)^,…,g^(*M*)^ are independent samples from an “importance sampling distribution” *Q*(·). With judicious choice of *Q* the standard deviation of this estimator, and hence the accuracy of the approximation, can be much improved compared with BF_naive_. Our importance sampling function is described in Text S1.

The third approach we consider is motivated by the simple idea of replacing unobserved genotypes at SNP *j* with their posterior mean, and computing a Bayes factor based on these posterior mean genotypes. To describe this approach more precisely, note that the Bayes factor for a typed SNP can be written as a function of a design matrix *X* (equation (8)). The approximation we consider is to replace *X* with its expected value, *X̅* (so in the second column of *X* each element g*_ij_* is replaced with E(g*_ij_*|*G*, *H*), and in the third column *I*(g*_ij_* = 1) is replaced with Pr(g*_ij_* = 1|*G*, *H*)) and to compute an approximate Bayes factor, BF_mean_, based on this expected design matrix:
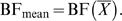
(11)Since the elements of *X̅* can be computed analytically (using either the PAC or cluster-based model) the approximation BF_mean_ is very quick to compute, reducing the number of Bayes factor evaluations by a factor of *M* (and thus, typically, reducing computation for each SNP by orders of magnitude, once imputation has been performed) compared with BF_naive_ and BF_IS_.

**Figure 2:**
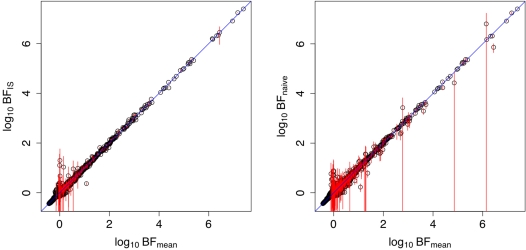
Graphs showing correspondence of BF_mean_ (*x*-axis) vs BF_IS_ (*y*-axis on left panel) and BF_naive_ (*y*-axis on right panel). In each case the diagonal blue line is *y* = *x*, and the vertical red lines indicate ±2 standard errors of the Bayes factor estimate (for example, on the left they run between log_10_(BF_IS_±2 standard errors)).

We compared the three approximations by applying them to the genome-scan genotype data on chromosome 22, described above, with phenotypes simulated under both null and alternative hypotheses (see next section for details). Bayes factors were computed by averaging over *σ_a_* = 0.05, 0.1, 0.2, 0.4 and *σ_d_* = *σ_a_*/4. For the sampling-based estimators BF_naive_ and BF_IS_ we used *M* = 1,000. Values of BF_mean_ were generally similar to both BF_IS_, and BF_naive_ (Figure 2), with the agreement with BF_IS_ being better (presumably because BF_IS_ has generally smaller standard error than BF_naive_; see red vertical bars on Figure 2). Furthermore, at SNPs where the values disagreed most strongly, the standard error of the Monte-Carlo estimators tended to be large (see red vertical bars on Figure 2), suggesting that at these SNPs the Monte-Carlo estimates may be unreliable. In summary, BF_mean_ appears to provide an adequate approximation to the Bayes factor, more accurate (for *M* = 1,000) than the naive Monte Carlo estimates used by [2],[3], and orders of magnitude quicker to compute.

To explain why, and under what circumstances, BF_mean_ provides an accurate approximation to the Bayes factor for *H*_1_ vs *H*_0_, note that BF_mean_ is, in fact, the Bayes factor for a different alternative hypothesis 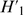,
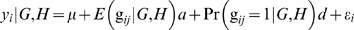
(12)where *ε_i_* are independent and identically distributed *N*(0,1/*τ*). The likelihood for this alternative hypothesis 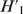 is very similar to the likelihood for *H*_1_ (Equation 7). In particular *Y_i_* has the same mean under 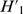 and *H*_1_. The differences between the two are that i) under 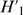, *Y_i_* is normal, whereas under *H*_1_ it is a mixture of three normals; and ii) under 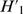 the variance of *Y_i_* is the same for each *i*, whereas under *H*_1_ the variance of *Y_i_* is larger for those *i* whose genotypes are less certain. These differences will be subtle unless *a* and *d* are large compared with 1/*τ* (since with small *a* and *d* the three components of the mixture will have similar means, making the mixture of normals very similar in shape to a normal, and ensuring that differences among individuals in variance of *Y_i_* are small). In particular, our empirical results suggest that, at least for priors with *σ_a_*<0.4, BF_mean_ generally provides an accurate approximation to the full Bayes factor. For different priors, using substantially larger values of *σ_a_*, we have seen examples where the accuracy of the approximation is much poorer (data not shown). However, in genome-wide association studies, where effect sizes of single SNPs tend to be small, the use of such large values of *σ_a_* will not generally be desirable or appropriate (although see next section).

We note that it would be relatively straightforward to develop an improved approximation to the Bayes factor for *H*_1_ by modifying 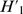 to account for the different variances across *i*. We do not pursue this here since BF_mean_ appears adequate for our purposes.

In subsequent sections we use BF_mean_ to approximate the Bayes factor. Motivated by the fact that BF_mean_ is the Bayes factor for 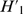 vs *H*_0_, we will also consider an analogous likelihood ratio statistic, Λ_mean_, defined to be the likelihood ratio statistic for 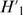 vs *H*_0_. The statistic Λ_mean_ has several practical advantages over Λ: it can be computed analytically (Text S2), which results in a moderate computation saving (a factor of around 10–20 in our implementation, although the exact saving will depend on the details of the numerical optimisation scheme used to obtain Λ); and Λ_mean_ can be easily obtained from standard regression software since 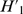 is a standard linear regression. (In terms of ranking SNPs in order of significance, Λ_mean_ is equivalent to the standard *F* statistic for this regression.)

### Choice of Test Statistic for Imputation-Based Analyses

In this section we use both theoretical arguments and simulation experiments to compare and contrast the use of Bayes factors vs *p* values from likelihood ratio statistics for testing untyped SNPs for association.

#### Theoretical Comparison

Several authors have pointed out advantages of Bayes factors over *p* values as measures of evidence (see for example, in this context, [2],[3],[8]). These advantages, which apply to both typed and untyped (imputed) SNPs, include many advantages of interpretation. For example, although *p* values have a straightforward interpretation in principle, they are notorious for being mis-interpreted in practice [16]. Further, the strength of the evidence represented by a particular Bayes factor tends to be less sensitive to context than the evidence represented by a particular *p* value. For example, the probability that a SNP with a Bayes factor of, say, 10^5^ is truly associated with the phenotype is the same whether the Bayes factor was computed from a sample of 100 or 10,000 individuals; whereas the probability that a SNP with a *p* value of, say, 10^−6^ is truly associated with phenotype will be different in these two settings. In addition it is often desirable, and straightforward, to combine (by averaging) Bayes factors computed under different assumptions (e.g. averaging over additive and dominant models for effects, and thus allowing for dominance while maintaining some of the benefits of simpler additive models). However, in terms of the limited question of simply *ranking* SNPs for association with phenotype, [17] shows that, under an additive model (no dominance), standard single-SNP *p* values provide the same ranking of SNPs as a particular Bayesian analysis. Specifically, these *p* values give the same ranking as is obtained from Bayes factors computed under the assumption that, under *H*_1_, the (prior) variance of effect sizes (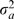 in this paper) is proportional to the variance of the maximum likelihood estimator (MLE) of the additive effect *a* at that SNP. Actually, [17] proves this result for a particular approximate Bayes factor and frequentist test statistic (the Wald statistic) in the setting of a logistic regression model; our Text S2 proves the analagous result for BF_mean_ and Λ_mean_ for the normal linear model. However, we expect that the result will hold more generally; see below for further discussion.

We now provide a practical interpretation of these results for typed and untyped SNPs.

For typed SNPs, the variance of the MLE is approximately proportional to 1/*f*(1−*f*), where *f* is the SNP minor allele frequency (MAF). Thus, as [17] points out, the result says that ranking SNPs by *p* values (from the Wald statistic, or Λ_mean_) can be thought of as making the implicit assumption that effect sizes tend to be larger for SNPs with a small MAF, and specifically that the expected square of the effect size is proportional to 1/*f*(1−*f*). Although we know of no strong evidence for this particular assumption, the general idea that rare SNPs may have larger effects is somewhat biologically plausible, providing a possible rationale for the use of *p* values in ranking typed SNPs for association.

For untyped SNPs, the variance of the MLE depends not only on the MAF, but also on the confidence with which the untyped SNP genotypes are imputed: the larger the uncertainty in imputed genotypes, the larger the variance of the MLE. Thus, in addition to the assumption for typed SNPs, ranking untyped (imputed) SNPs by *p* values (from the Wald statistic or Λ_mean_) can be thought of as making the implicit assumption that *the harder a SNP is to impute*, *the larger its expected effect size*. This assumption seems hard to argue for on any grounds, and we believe that this explains the results in simulations below, where Λ and Λ_mean_ show slightly poorer performance than Bayes factors that do not make this assumption.

#### Empirical Comparisons

To complement the above theoretical arguments, we compared methods empirically, using the real chromosome 22 genotype data described above and simulated phenotype data. To make the comparison as focussed as possible we compare results from the likelihood ratio statistics, Λ and Λ_mean_, with the Bayes factor BF_mean_ based on the implicit prior assumed by the likelihood ratio statistic. That is, we used a prior in which the expected square of the effect size, 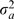, is proportional to 1/*f*(1−*f*) (where *f* is the SNP MAF, estimated from the panel data). Specifically we averaged Bayes factors over four values, 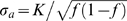 with *K* = 0.05,0.1,0.2,0.4. (Note that for the small proportion of SNPs with very small *f* this produces very large values of *σ_a_* for which the mean genotype approximation that we are using may not provide a very accurate approximation to the full Bayes factor. This presumably reduces power slightly compared with what could be achieved using a better approximation.) All statistics were computed under a pure additive model (*d* = *σ_d_* = 0).

The key point here is that the priors for the Bayesian approach were chosen so that, under the theory outlined above, the Bayesian and frequentist test statistics will provide the same rankings for typed SNPs. Thus any difference between the methods in ranking SNPs must be due to differences in the treatment of untyped imputed SNPs.

We simulated phenotype data under two scenarios. The first scenario makes the assumption (also made by the testing methods) that the additive effect at the causal SNP depends on MAF 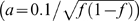. The second scenario assumes that the effect size is constant, independent of MAF (*a* = 0.2). These values of *a* provide modest power to detect effects with the sample size (*n* = 675) we use here. Both scenarios assumed no dominance, *d* = 0, consistent with the assumptions made in computing the statistics. For each value of *a* we simulated 1100 sets of phenotypes. Each set of phenotypes was simulated by selecting a masked SNP to be the “causal” variant, and then generating phenotype values according to equation 6. This essentially creates 1100 independent genome scan datasets, for chromosome 22 only, each containing a single causal variant.

We applied imputation-based association mapping to each dataset, using the HapMap CEU individuals as a panel, and computing test statistics (BF_mean_, Λ and Λ_mean_) for each typed and untyped SNP. To examine effects of lower average imputation accuracy (and confidence) we repeated this experiment using the YRI individuals as a panel. In each case the causal variants were assumed to be untyped (i.e., the genotypes at the causal variant were assumed to be unknown, and were imputed along with all other untyped SNPs). To compute Λ we used numerical maximisation (Nelder-Mead) to maximise the numerator, with initial parameter values obtained from maximising the alternative likelihood (12). The computation for one simulated phenotype (34083 test statistics) for BF_mean_, Λ and Λ_mean_ take 20 seconds, 229 seconds, and 10 seconds respectively, after imputed genotype probabilities had been computed.

**Figure 3:**
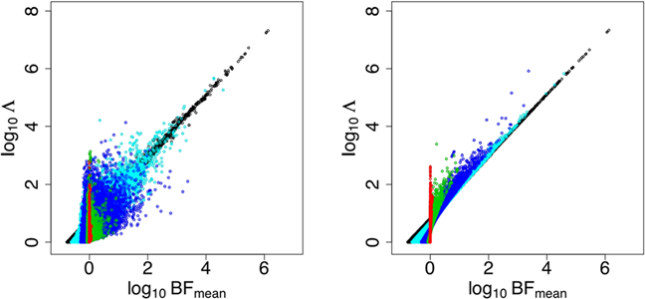
Graph showing correspondance between log_10_(BF_mean_) values and Likelihood ratio statistics Λ (left) and Λ_mean_ (right). Each point represents one SNP-phenotype combination, colored according to average confidence in imputed genotypes. Specifically, the SNPs were colored according to the value of *r*, defined to be the ratio of the variance of the (posterior mean) genotypes and the expected variance if the SNP were typed, calculated from the MAF assuming Hardy-Weinberg equilibrium (2*f*(1−*f*)). This scaling ensures that for typed and confidently-imputed SNPs *r*≈1, whereas for SNPs with low average confidence *r* will be close to 0. Colors indicate ranges of values of *r*: red *r*∈(0,0.001]; green *r*∈(0.001,0.01]; blue *r*∈(0.01,0.1]; cyan *r*∈(0.1,0.5]; black *r*>0.5.

Figure 3 illustrates the relationships between BF_mean_ and Λ,Λ_mean_, and how they depend on imputation confidence. (The relationships with Λ and Λ_mean_ are qualitatively similar, but the relationship with Λ_mean_ is cleaner because of the mathematical relationship between BF_mean_ and Λ_mean_ given in Text S2). Where SNPs are easy to impute, with high confidence genotypes (black in Figure 3), the Bayes factor and Likelihood ratio statistics have an almost monotonic relationship, as predicted by the theory. For difficult-to-impute SNPs, with low confidence genotypes, (e.g., red in Figure 3) the qualitative behaviour of the statistics differs. The Bayes factor tends to take values close to 1 (log(BF_mean_) close to 0), with the variance about 1 being smallest for those SNPs with the lowest confidence genotypes. This reflects the fact that these difficult-to-impute SNPs tend to be relatively uninformative regarding the null vs alternative hypotheses. In contrast, by design, the likelihood ratio statistics have approximately the same distribution (under the null) for both poorly-imputed and well-imputed SNPs. The practical effect of this is that, when testing difficult-to-impute SNPs, the Bayes factor is less likely to take a large value by chance than is the likelihood ratio statistic, and so difficult-to-impute SNPs are less likely to produce false positives by chance when using the Bayes factor than using the likelihood ratio statistic.

To quantify the practical impact of this difference between the approaches, we compared the power of the BF and LR to distinguish true positive vs false positive signals, and compared both these approaches with not performing imputation. Since large test statistic values tend to cluster together (due to LD among nearby SNPs), and since we want to compare methods that are testing different sets of SNPs (imputation vs no imputation), we took the approach of defining regions, rather than individual SNPs, as the unit of “discovery”. Specifically, we divided the chromosome into adjoining 200 kb regions, chosen in such a way that one region is centered on the causal variant, and scored each region as a “discovery” if the largest Bayes factor (or the largest value of Λ) in that region exceeded some threshold (*T*, say). Each discovery was considered a “true discovery” if it contained the causal variant, and a “false discovery” if it did not. (To reduce the effects of potential false positives due to LD among regions, we ignored the regions either side of the region containing the causal SNP when scoring discoveries as true or false; we also checked that our qualitative conclusions were insensitive to precise choice of region size.) Note that, as threshold *T* decreases, the number of true discoveries and false discoveries necessarily increases. Note also that, since the imputation approach involves computing test statistics for both typed and untyped SNPs, it will inevitably increase the number of both true and false discoveries (for given *T*) compared with testing typed SNPs only. To make a fair comparison between different approaches we therefore focus on comparing the number of true discoveries, for a given number of false discoveries, by plotting the number of true vs. false discoveries as *T* varies. Since the relative performance of different methods was similar for the two simulation scenarios, we show results averaged across the scenarios.

**Figure 4:**
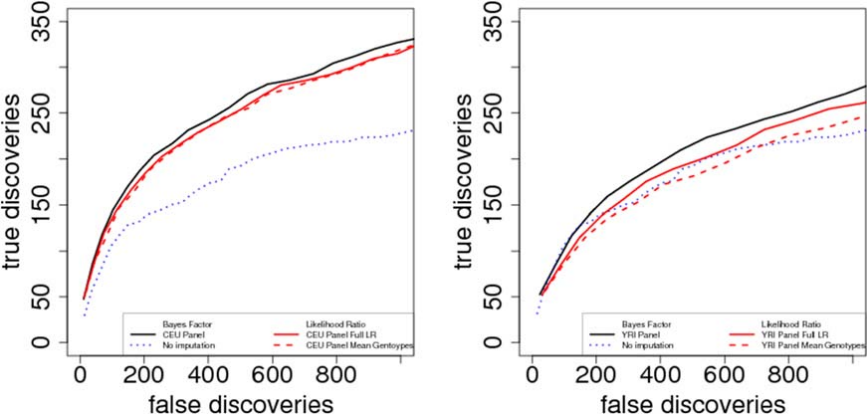
Effects of different test statistics on power to detect associations. Each line shows the trade-off between true and false discoveries when using Bayes factors (black lines) or likelihood ratio test statistics Λ (red solid lines) and Λ_mean_ (red dashed lines), as threshold for declaring an association is varied. In each setting the Bayes factor produces as good, or better, performance than the likelihood ratio test (black lines are above the corresponding red lines). Best performance is obtained using CEU panel, which is well-matched to the sample and produces a low imputation error rate (left; imputation error rate 6.2%). Larger increases in imputation error rate, obtained when using YRI panel that is not well-matched to sample, produce a notable reduction in performance (right; imputation error rate 25%). However, even with a high imputation error rate, using the Bayes factor as a test statistic gives better results than no imputation (blue dotted lines in both panel).

The results (Figure 4) confirm that the BF has an advantage compared with the likelihood ratio statistics: for any given number of true positives, the BF produces fewer false positives. This advantage is small for the CEU panel, and larger for the YRI panel where there are more poorly-imputed SNPs with low confidence genotypes. For the YRI panel the full likelihood ratio performed better than Λ_mean_, presumably because the approximate model on which Λ_mean_ is based is a worse approximation for harder-to-impute SNPs.

#### Summary of Comparisons of Bayes Factor and Likelihood Ratio

In summary, when testing imputed variants for association with a normal phenotype, we found that using a Bayes factor to rank SNPs for strength of evidence of a association gave better performance than using the Likelihood ratio statistics. Theoretical arguments suggest that this gain in performance may be explained by viewing the Likelihood ratio statistics as making an implicit, and unnatural assumption: that difficult-to-impute variants tend to have larger effect sizes. Consistent with this, we found that the quantitative difference in performance depends on the fraction of difficult-to-impute SNPs, and is larger when many SNPs have low-confidence imputed genotypes. Thus, in practice, the difference between the methods might have greater importance in imputation applications involving harder-to-impute rare variants (e.g. where the panel data are resequencing data, rather than genotype data–a scenario that may be more common in the near future with gains in sequencing technology).

It is natural to ask whether the advantage of the Bayes factor over the likelihood ratio statistics in this setting (of normal phenotype) also transfers to other settings, and to other frequentist test statistics. In fact we believe there are several reasons to expect it will apply quite generally. For example, regarding the transfer to settings other than normal phenotypes, the theoretical results from [17] apply to logistic regression models. Further, Wakefield's results are based on an approximate Bayes factor that itself is based on asymptotic normality of the maximum likelihood estimate, and it seems quite probable that similar results can be obtained in other settings where the maximum likelihood estimate is asymptotically normal.

Regarding the extension to other frequentist test statistics, we note that many test statistics in common use (e.g. the *F* statistic in standard regression) are asymptotically equivalent to the likelihood ratio test statistic. Furthermore, we view the root cause of the loss of power of the likelihood ratio statistics compared with the Bayes factor as being due to the fact that under the null, by design, the likelihood ratio statistics have the same distribution for all SNPs. (In contrast, under the null, the Bayes factors for difficult-to-impute SNPs tend to be closer to 1 than for other SNPs–Figure 3–reflecting the fact that there is little information in these SNPs to distinguish the null and alternative hypotheses.) This design feature limits the ability of Λ and Λ_mean_ to respond to differences in informativeness among SNPs, and in particular it causes them to occassionally produce, by chance, high ranks for difficult-to-impute SNPs. We therefore expect other test statistics that have this feature of having a fixed distribution under the null (which would include most conventional frequentist test statistics used for single-SNP analyses, including chi-square statistics for case-control studies, and the score test in [3]) to suffer similar problems. Note that, based on this insight, one could attempt to design frequentist procedures that circumvent this problem. For example, one could rank SNPs using the Bayes factors we present here, and then assess significance of the largest Bayes factor by permutation, providing a *p* value for the global null hypothesis of no association between the genotypes and phenotypes, and thus controlling the family-wise type I error rate. Alternatively, and preferably, one could try to design a similar procedure that ranks SNPs by their Bayes factor, and then controls the False Discovery Rate [18]. A simpler, and more *ad hoc* fix, would be to simply avoid testing SNPs that are difficult to impute; this may well provide an effective practical solution, although the inevitable arbitrariness in deciding which SNPs are sufficiently well-imputed to test seems inherently unsatisfying.

We emphasise that we do not view the gain in power discussed here as the only, or indeed the strongest, argument for applying Bayesian methods: we have focussed on it because it is an issue specific to imputation analyses, and one that has not previously been discussed. We also emphasise that the prior we used here, where effect size depends on minor allele frequency, and with no dominance, was adopted purely to facilitate comparisons with the likelihood ratio statistics. If effect sizes are independent of (or only weakly dependent on) MAF, or if some loci exhibit dominant effects, then increased power should be obtained from a Bayesian analysis that incorporate these factors. A strength of the Bayesian approach is its ability to incorporate this type of information where it is available, and to average over assumptions where good data are not available. For example, since there is little direct data on relationship between the MAF and effect size, there is an argument for averaging Bayes factors obtained under both the independence and dependence assumptions; similarly, there is an argument for averaging Bayes factors over different amounts of dominance, for example by setting *σ_d_*>0 (of course, any *particular* value of *σ_d_* is going to be somewhat arbitrary, but setting it to be small seems preferable to assuming no dominance).

### Effect of Imputation Accuracy on Power

Besides comparing the Bayesian and frequentist approaches to inference, the results in Figure 4 also illustrate that, at least when using the Bayes factor to test imputed SNPs, the imputation-based approach is relatively robust to very poor imputation accuracy: even with very high average imputation error rate (25% error rate with YRI panel) the imputation-based method using the Bayes factor as a test statistic performs better than testing typed markers only. This is reassuring for the use of imputation in studies where no well-matched panel is available. We also examined the effect of smaller changes in imputation accuracy (imputation error rates in range 6.2% to 7.3%). As might be expected, such small changes in imputation error rate produce correspondingly small changes in performance (comparable in magnitude to the difference between BF_mean_ and Λ for the CEU panel in Figure 4; data not shown).

### Binary Phenotypes

**Figure 5:**
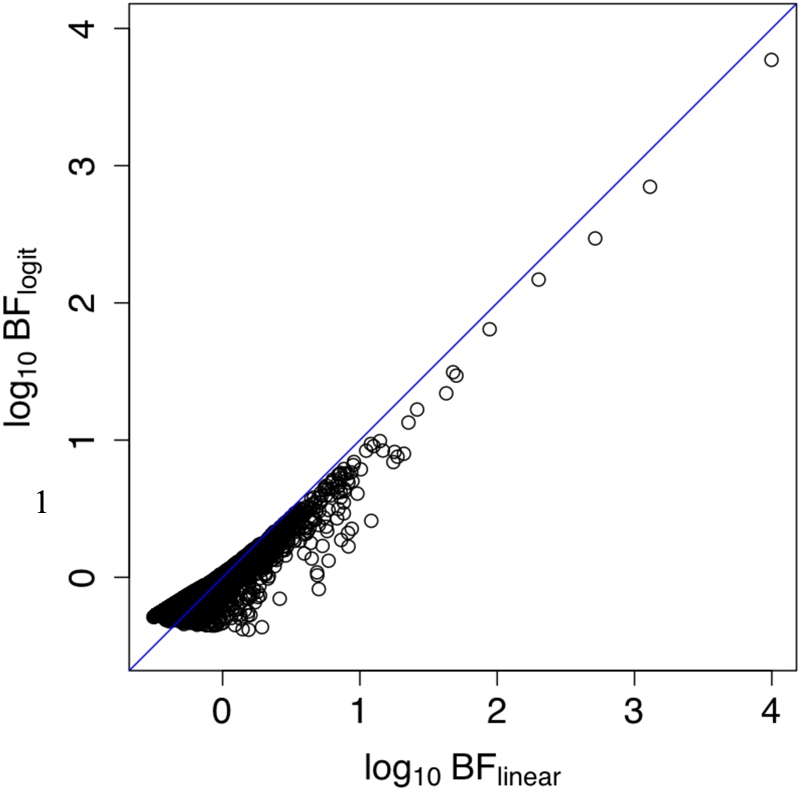
The x-axis is log_10_ (*BF*) of linear model and y-axis is log_10_ *BF* of logistic model. The blue line is *x* = *y*.

While we have focussed here on testing quantitative phenotypes for association with genotypes, all of the key results should be expected to also apply to binary (0/1) phenotypes. Indeed, although the natural way to analyse a binary trait is via a logistic, rather than a linear, regression, for the small effect sizes that are typical in genetic studies the two approaches to analysis might be expected to produce similar results (e.g. see [19], p. 18). To examine this further, we simulated binary phenotypes *Y*′, by taking the quantitative phenotypes *Y* simulated above, and setting 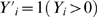. We then compared the Bayes factors for these binary phenotypes under the linear model (using the mean genotype approximation based on (8)), and the logistic model [using the Laplace method to approximate necessary integrals, as was done in 9; see Text S3]. The results (Figure 5) show a strong correspondence between Bayes factors based on the two different models, supporting the idea that results obtained here for quantitative traits will apply also to binary traits.

## Discussion

In summary, we have addressed a number of practical issues that arise in implementing imputation-based association mapping for a quantitative trait. Key findings include: i) when using the model of [7] to impute untyped SNPs, fitting the model to only the panel produces substantially improved imputation accuracy compared with fitting the model to all data; ii) although imputation accuracy is affected by mismatches in genetic background between panel and study samples, imputed genotypes can be accurate provided the panel contain at least some samples that are representative of the study sample; iii) when computing Bayes factors based on imputed genotypes, simply replacing the imputed genotypes with their posterior mean produces a good approximation to a full analysis; iv) when ranking imputed SNPs for association, in our simulations Bayes factors produced better rankings than Likelihood ratio statistics; our explanation for this, based on theoretical arguments, is that the Bayes factors take better account of the different amounts of information in different imputed SNPs; v) The power of Bayesian imputation-based association mapping is relatively robust with respect to imputation accuracy: even when average imputation accuracy is low, imputation-based analysis can increase power compared with testing typed SNPs only. More generally, since in our simulations small differences in imputation accuracy had only a small effect on power, we conclude that choice of imputation method may ultimately matter less than what is done with the imputed genotypes. Thus, when selecting software to use in performing imputation-based analyses, it seems important to consider the range of analyses that can be performed, as well as the imputation method used. For example, our software, BIMBAM, can perform not only the single-SNP tests based on Bayes factors described here, but also multi-SNP analyses of individual genes or regions; and it can easily exploit any (unphased) resequencing data that may be available on regions of interest. All these features can increase power to detect, and ability to explain, associations [2].

While our findings are based on use of a particular imputation method, in the particular context of association mapping of a quantitative phenotype, all of them (except perhaps i) above) are likely to apply more generally. For example, the PAC model used for imputation in [3] has many similarities with the one we use here, including the fact that imputation is essentially performed by modelling each sampled haplotype as a mosaic of template haplotypes (in the PAC model these template haplotypes are the panel haplotypes, where in the cluster-based model these template haplotypes are estimated from the panel haplotypes, and are in some sense a summary of the panel haplotypes). As such, finding (ii) regarding the impact of mismatches in genetic background between panel and cohort individuals are also likely to apply to this method. Similarly, findings (iii–vi) seem likely to apply not only to quantitative traits, but also binary traits, or case-control studies, particularly given the correspondence between the Bayes factors based on linear and logistic models, in Figure 5.

Although imputation-based analyses involve considerably more computation than simply testing typed SNPs, these analyses are nevertheless now practical with very modest computational resources. For example, using the methods we describe here (and particularly findings i) and iii) above), implemented in the software package BIMBAM, analysing the whole of chromosome 22, in 675 individuals, with *E* = 5 and *K* = 10 takes just under an hour on a single Mac PRO desktop computer (with 3.0 GHz CPU and 8G memory, although memory used for simulations here was less than 1G).

Besides the gain in computational convenience, the effectiveness of Bayes factors based on posterior mean genotypes also has important implications for sharing and combining data across studies. Specifically, we have in mind situations where, for political, ethical, or other reasons, sharing individual-level genotype and phenotype data among investigators working on similar studies is more difficult than sharing summary-level data on each SNP. In these cases, a simple approach is to share and compare Bayes factors (or *p* values) for each SNP. However, better summaries of the data can be easily shared, to allow more powerful subsequent analyses. Specifically, for a quantitative trait, for a typed SNP the Bayes factor (8) depends on the phenotype and genotype data only through the matrices *X^t^X*, *X^t^Y* and the number *Y^t^Y*. Similarly, for an imputed SNP, BF_mean_ depends only on *X̅* *^t^X̅*, *X̅* *^t^Y* and *Y^t^Y*. These three quantities represent only summary level data (for example, in the case of the observed SNP they are essentially equivalent to knowing the number of individuals in each genotype class, and the within-genotype-class means and standard deviations of the phenotype). Further, if these three quantities are known for multiple studies, they can be computed for the combined study. Specifically, if *X_s_* and *Y_s_* denote, respectively, the design matrix and phenotype vector for study *s* (*s* = 1,…,*S*), and *X* and *Y* denote the combined design matrix and phenotype vector for the combination of data across all *S* studies, then it is straightforward to show that
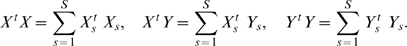
(13)Since *X^t^X*, *X^t^Y* and *Y^t^Y* suffice to compute the Bayes factor for the combined study, this demonstrates that, from appropriate summary-level data from each study, one can compute the Bayes factor BF_mean_ for each SNP *as if one had possession of the combined data across all studies*. (Note that the same summary data also suffice to compute Λ_mean_, allowing frequentist inference for the joint data also to be performed without sharing individual-level data.)

Of course, combining results across studies can present many challenges, including differential inclusion criteria or phenotype definitions; differential genotyping biases, e.g. due to differential DNA quality [20]; and different phenotype distributions and/or genetic backgrounds within different studies. Some of these problems may be easier to solve than others (e.g. to minimize this last problem we advocate quantile normalizing the phenotype values within each study, to an *N*(0,1) distribution, prior to computing summary statistics.) However, our results do provide a framework for combining results across studies when these other challenges can be surmounted. Our software BIMBAM includes an option to output and input summary statistics of this form, allowing multiple investigators to easily perform a combined imputation-based analysis of multiple data sets, without sharing individual level data among groups.

The methods described here, like those from [3], are based on a prospective likelihood. However, they have already been applied to other designs, such as case-control studies [9], where use of a retrospective likelihood would be more appropriate. We now consider the validity of this approach. For case-control studies, with observed covariates, the correspondance between Bayesian analyses based on prospective and retrospective likelihoods is derived by [21]. In brief, they show that the Bayesian analysis based on a prospective likelihood is equivalent to a particular Bayesian analysis using the retrospective likelihood, provided that the prospective analysis uses an (improper) uniform prior on the baseline log-odds of the disease (e.g. log-odds of disease for genotype 0). [This is the prior implemented in our software, and it can also be shown (MS, unpublished data) that the approximate BF from [17] can be derived using this prior. This was not the prior used in [9], although it is unclear how much this matters in practice.] This result justifies the use of Bayesian methods based on a prospective model, with an appropriate prior on the baseline log-odds, for analysing typed SNPs in case-control studies. The result does not apply directly to unobserved covariates, and hence does not apply directly to untyped SNPs. However, we note that i) if a SNP is easy to impute, with high confidence genotypes, then the result seems likely to hold approximately, since it is almost as if the SNP is observed; ii) if a SNP is difficult to impute, with very low confidence genotypes, the Bayes factors from prospective and retrospective analyses will both be close to 1, since there will be little information regarding association at such SNPs. Thus, for SNPs that are very easy or very difficult to impute the prospective analysis will approximately agree with a retrospective analysis. This gives grounds for optimism that results from applying these prospective methods to case-control data will not be generally misleading. The situation is slightly less clear for data obtained by genotyping individuals that are at the extremes of a quantitative phenotype distribution (e.g. [22]), since the [21] result applies to binary phenotypes. Thus, although we speculate that direct prospective analyses of the observed quantitative phenotypes will be generally satisfactory here also, it might be prudent to also analyse such data treating the two extremes as binary (case-control) phenotypes.

## Supporting Information

Text S1 Importance sampling.
(0.06 MB PDF)

Text S2 Relationship between BF_mean_ and Λ_mean_.
(0.06 MB PDF)

Text S3 Laplace method to approximate Bayes factors for Logistic Regression.
(0.06 MB PDF)
