# Supplementary material for: Practical Issues in Imputation-Based Association Mapping
Source: PLoS Genet. 2008 Dec 5;4(12):e1000279. doi: 10.1371/journal.pgen.1000279 (PMC2585794; doi:10.1371/journal.pgen.1000279)
Supplement: Text S1 — Importance sampling. (0.06 MB PDF) [file pgen.1000279.s001.pdf]

Text S1: Importance Sampling

Here we describe the importance sampling distribution used to approximate the Bayes factors for Figure 2 in the main text. Intuitively, the aim of importance sampling is to avoid the large standard errors that can plague the naïve estimate by concentrating more of the samples on genotype configurations that produce large Bayes factors. The optimal choice of importance sampling distribution is easily shown to be

$$Q_{opt}(g_{\cdot j}) = \Pr(g_{\cdot j}|G, H, Y, \text{SNP } j \text{ is QTN}) \propto \Pr(g_{\cdot j}|G, H)p(Y|g_{\cdot j}, \text{SNP } j \text{ is QTN}). \quad (1)$$

Here we use "SNP j is QTN" as shorthand for "SNP j is associated with phenotype". Indeed, for this choice of Q the estimator has 0 standard error, and hence provides a perfect approximation to (5) in the main text. This optimal distribution cannot itself be used because the constant of proportionality cannot be computed. Instead, we attempt to approximate this distribution as follows.

Let $\bar{X}$ denote the expectation of the design matrix X used in the computation of BF_{mean}. Let $\hat{\beta}$ denote the maximum *a posteriori* value for $\beta = (\mu, a, d)$ under the alternative hypothesis H_1' (12) in the main text:

$$\hat{\beta} = (\nu^{-1} + \bar{X}^t\bar{X})^{-1}\bar{X}^tY. \quad (2)$$

We use this estimate of β to define an importance sampling function $\tilde{Q}$, by replacing $p(Y|g_{\cdot j}, \text{SNP j is QTN})$ in (14) in the main text with an estimate of this quantity based on $\hat{\beta}$:

$$\begin{aligned} \tilde{Q}(g_{\cdot j}) &\propto \Pr(g_{\cdot j}|G, H)p(Y|g_{\cdot j}, \hat{\beta}, \text{SNP } j \text{ is QTN}) \\ &\propto \prod_i \Pr(g_{ij}|G, H)p(Y_i|g_{ij}, \hat{\beta}, \text{SNP } j \text{ is QTN}). \end{aligned} \quad (3)$$

Note that this product form for $\tilde{Q}$ implies that the genotypes of different individuals are independent. As a result, the constant of proportionality for $\tilde{Q}$ can be computed (by normalising each individual's genotype probabilities to sum to 1).

Intuitively, like Q_{opt}, $\tilde{Q}$ places more mass on genotype configurations that give a higher likelihood for the phenotype data Y, but it achieves it in an *ad hoc* way by first estimating β, and then adjusting the mass on each configuration assuming the estimated β is correct.

In importance sampling it is generally considered helpful to have an importance sam-

pling function with "long tails", in that it is not too concentrated on particular parts of the space. Because $\tilde{Q}$ ignores the fact that there may be considerable uncertainty in the actual value of β, there is a danger that $\tilde{Q}$ may place too much of its mass on configurations that are consistent with the particular point estimate obtained, at the expense of other configurations. To help guard against this we use the approach of [1], mixing $\tilde{Q}$ with a longer-tailed distribution, specifically the distribution of genotypes unconditional on the phenotype data, $\Pr(g_{\cdot j}|G, H)$. Thus the importance sampling distribution used here is

$$Q(g_{\cdot j}) = 0.1 \Pr(g_{\cdot j}|G, H) + 0.9\tilde{Q}(g_{\cdot j}). \tag{4}$$

References

[1] Hesterberg, T. C. (1995). Weighted average importance sampling and defensive mixture distributions. *Technometrics*, **37**, 185–194.
