# Supplementary material for: Practical Issues in Imputation-Based Association Mapping
Source: PLoS Genet. 2008 Dec 5;4(12):e1000279. doi: 10.1371/journal.pgen.1000279 (PMC2585794; doi:10.1371/journal.pgen.1000279)
Supplement: Text S2 — Relationship between BFmean and Λmean. (0.06 MB PDF) [file pgen.1000279.s002.pdf]

Text S2: Relationship between $\mathrm{BF}_{\mathrm{mean}}$ and Λ_{mean}

Here we develop an expression for the relationship between the Bayes factor $\mathrm{BF}_{\mathrm{mean}}$ and the likelihood ratio statistic Λ_{mean}, under an additive model with $d = 0$ and a fixed value of the prior variance of a, σ_a. (In the main paper we use a Bayes factor that averages over several values of σ_a, and allows for some dominance, as in [1]. This is different from, though highly correlated with, the Bayes factor considered here.)

Under an additive model ($d = 0$) the model (12) in the main text can be written as $Y \sim N(\bar{X}\beta, 1/\tau)$, where $\bar{X}$ is the $n \times 2$ design matrix $(\mathbf{1}, \bar{g})$ whose first column is all 1s, and second column contains the expected genotype values for each individual. Using the prior D2 from [1] the Bayes factor for the alternative ($a \neq 0$) vs the null ($a = 0$) is:

$$\mathrm{BF}_{\mathrm{mean}} = \frac{(\alpha n)^{1/2}}{\det(\Omega)^{1/2}} \left(\frac{Y^t Y - Y^t \bar{X} \Omega^{-1} \bar{X}^t Y}{Y^t Y - n\bar{Y}^2} \right)^{-n/2} \tag{1}$$

where for notational convenience we have written α for σ_a^{-2}, and $\Omega = \left(\begin{smallmatrix} 0 & 0 \\ 0 & \alpha \end{smallmatrix}\right) + \bar{X}^t \bar{X}$. If we let V denote n times the sample variance of g, $V = (g^t g - n\bar{g}^2)$, then $\det(\Omega) = n(\alpha + V)$, $\det(\bar{X}^t \bar{X}) = nV$, and

$$\begin{aligned} \Omega^{-1} &= \frac{1}{\det(\Omega)} \left[\det(\bar{X}^t \bar{X}) (\bar{X}^t \bar{X})^{-1} + \left(\begin{smallmatrix} \alpha & 0 \\ 0 & 0 \end{smallmatrix}\right) \right] \\ &= \frac{V}{\alpha + V} \left[(\bar{X}^t \bar{X})^{-1} + \left(\begin{smallmatrix} \alpha/nV & 0 \\ 0 & 0 \end{smallmatrix}\right) \right]. \end{aligned} \tag{2}$$

Now let RSS_0 and RSS_1 denote the residual sum of squares for the maximum likelihood estimates under the null and alternative hypotheses:

$$\begin{aligned} \mathrm{RSS}_0 &= Y^t Y - n\bar{Y}^2 \\ \mathrm{RSS}_1 &= Y^t Y - Y^t \bar{X} (\bar{X}^t \bar{X})^{-1} \bar{X}^t Y. \end{aligned} \tag{3}$$

Taking the log of (1), and substituting, we obtain

$$\begin{aligned} \log(\mathrm{BF}_{\mathrm{mean}}) &= 0.5 \log[\alpha/(\alpha + V)] - (n/2) \log \frac{\alpha/(\alpha + V)\mathrm{RSS}_0 + V/(\alpha + V)\mathrm{RSS}_1}{\mathrm{RSS}_0} \\ &= 0.5 \log[\alpha/(\alpha + V)] - (n/2) \log \left[1 - \frac{V}{\alpha + V} \frac{\mathrm{RSS}_0 - \mathrm{RSS}_1}{\mathrm{RSS}_0} \right]. \end{aligned} \tag{4}$$

Note also that the log-likelihood ratio statistic is given by

$$2 \log \Lambda_{\text{mean}} = n \log[\text{RSS}_0/\text{RSS}_1] = n \log[1 - (\text{RSS}_0 - \text{RSS}_1)/\text{RSS}_1]. \tag{5}$$

Four observations come directly from these expressions for BF_{mean} and Λ_{mean}:

- For fixed V, both BF_{mean} and Λ_{mean} are monotone decreasing in $(\text{RSS}_0 - \text{RSS}_1)/\text{RSS}_1$. Hence, among SNPs with the same value of V, BF_{mean} and Λ_{mean} will produce the same ranking of SNPs.

- If $\alpha = kV$ (so that for each SNP the prior variance on a is chosen to be inversely proportional to the V for that SNP) then $\log(\text{BF}_{\text{mean}})$ and Λ_{mean} produce the same ranking of SNPs.

- For fixed V, if $(\text{RSS}_0 - \text{RSS}_1)/\text{RSS}_1$ is small (which it will be in our applications, where effect sizes are small), then both $\log(\text{BF}_{\text{mean}})$ and Λ_{mean} are approximately linear in $(\text{RSS}_0 - \text{RSS}_1)/\text{RSS}_1$, because $\log(1 - x) \approx -x$ when x small. This explains the approximately linear relationships evident in Figure 3 in the main text.

- For fixed $(\text{RSS}_0 - \text{RSS}_1)/\text{RSS}_0$, and fixed α, $\text{BF}_{\text{mean}} \to 1$ as $V \to 0$. Thus, compared with Λ_{mean}, BF_{mean} down-weights SNPs with small V.

References

[1] Servin, B. and Stephens, M. (2007). Efficient multipoint analysis of association studies: candidate regions and quantitative traits. *PLoS Genetics*, **3**.
