# Supplementary material for: Practical Issues in Imputation-Based Association Mapping
Source: PLoS Genet. 2008 Dec 5;4(12):e1000279. doi: 10.1371/journal.pgen.1000279 (PMC2585794; doi:10.1371/journal.pgen.1000279)
Supplement: Text S3 — Laplace method to approximate Bayes factors for Logistic Regression. (0.06 MB PDF) [file pgen.1000279.s003.pdf]

Text S3: Laplace method to approximate Bayes factors for Logistic Regression

We now detail the Laplace method that we use to approximate the Bayes factor for a binary phenotype $Y = (y_1, \dots, y_n)$, which we assume to be modeled by a logistic regression:

$$\log \frac{\Pr(y_i = 1)}{\Pr(y_i = 0)} = \langle x_i, \beta \rangle$$

where $x_i = (1, g_i, 1(g_i = 1))$ is the i-th row of the design matrix X; $\beta = (\mu, a, d)$ is the vector of effect parameters; and $\langle x_i, \beta \rangle$ denotes the inner product $x_i^t \beta$. Then

$$p_i := \Pr(y_i = 1) = \frac{e^{\langle x_i, \beta \rangle}}{1 + e^{\langle x_i, \beta \rangle}}, \tag{1}$$

and the log-likelihood is given by

$$\begin{aligned} l(\beta | X, Y) = & \sum_i \big(y_i \log p_i + (1 - y_i) \log (1 - p_i) \big) \\ = & \sum_{i=1}^{n} y_i \langle x_i, \beta \rangle - \sum_{i=1}^{n} \log (1 + e^{\langle x_i, \beta \rangle}). \end{aligned} \tag{2}$$

Under the null hypothesis we assume that $a = d = 0$, and the prior on μ is $N(0, \sigma_\mu^2)$. That is,

$$p_0(\mu) = \frac{1}{2\pi\sigma_\mu} \exp\Big(-\frac{\mu^2}{2\sigma_\mu^2} \Big). \tag{3}$$

Under the alternative hypothesis, we assume the prior on β is $N(0, \nu)$, where ν is a diagonal matrix with diagonal elements $(\sigma_\mu^2, \sigma_a^2, \sigma_d^2)$. That is,

$$p_1(\beta) = (2\pi)^{-\frac{3}{2}} \frac{1}{\sigma_\mu \sigma_a \sigma_d} \exp\Big(-\frac{\mu^2}{2\sigma_\mu^2} - \frac{a^2}{2\sigma_a^2} - \frac{d^2}{2\sigma_d^2} \Big). \tag{4}$$

The Bayes factor is given by

$$\mathrm{BF} = \frac{\int l(\beta | X, Y) p_1(\beta) d\beta}{\int l(\mu | X, Y) p_0(\mu) d\mu}. \tag{5}$$

We approximate each of the integrals by the Laplace method,

$$\int e^{f(\beta)} d\beta \approx (2\pi)^{\frac{k}{2}} |H_{\beta^*}|^{-\frac{1}{2}} e^{f(\beta^*)} \tag{6}$$

where k is the dimension of the integral being approximated, β^* is the value at which f attains its maximum, and $|H_{\beta^*}|$ is the absolute value of the determinant of the Hessian matrix of f evaluated at β^*.

Under the alternative, $k = 3$, $f(\beta) = l(\beta|X, Y) + \log p_1(\beta)$. The Hessian H is given by

$$H = -(X^t W X + \nu) \tag{7}$$

where W is the diagonal matrix with $W_{ii} = p_i(1 - p_i)$. We obtained β^* by numerical optimization, using the Fletcher-Reeves conjugate gradient algorithm implemented in the GNU Scientific Library.

Under the null, $k = 1$, $f(\mu) = l(\mu|X, Y) + \log p_0(\mu)$, and the Hessian is trivially obtained.
